# Effects of *Spartina alterniflora* Extract on Growth Performance and Flavor Quality in Mud Crab (*Scylla paramamosain*)

**DOI:** 10.3390/foods14244176

**Published:** 2025-12-05

**Authors:** Yuanyuan Fu, Ao Li, Peng Gao, Yanrong Li, Lei Liu, Xuedi Du, Xiaojing Dong, Congying He

**Affiliations:** 1 Ningbo Institute of Oceanography, Ningbo 315000, China; fuyuan0101@163.com (Y.F.);; 2 Animal Science and Technology College, Yangzhou University, Yangzhou 225000, China; 15050730638@163.com (A.L.);; 3 School of Marine Sciences, Ningbo University, Ningbo 315832, China

**Keywords:** aquafeed additives, umami compounds, fatty acid, GC-MS

## Abstract

The steady rise in living standards has created a growing market demand for aquatic products with superior flavor profiles and enhanced nutritional value. To address this need, the present study investigates the effects of dietary supplementation with Spartina alterniflora (SA) extract on growth performance and muscle quality parameters in mud crabs (*Scylla paramamosain*). In a 63-day feeding trial, 150 juvenile crabs received experimental diets containing 0% (control), 0.05%, 0.1%, 0.15%, or 0.2% SA extract. The results showed that optimal growth enhancement was achieved with 0.15% supplementation. Flavor analysis revealed dose-dependent improvements in umami characteristics. Supplementation at ≥0.1% significantly increased the concentrations of key umami compounds; their taste activity values; and equivalent umami concentrations. Fatty acid analysis showed that extract supplementation modulated lipid composition, increasing eicosapentaenoic acid while decreasing docosahexaenoic acid content, with the n-3/n-6 ratio remaining stable across all treatments, except in the 0.2% group. Volatile compound analysis demonstrated that ≥0.1% supplementation enhanced aldehyde production, improving flavor profiles. Interestingly, while 0.1–0.15% supplementation produced predictable flavor modifications, the 0.2% group showed atypical responses in flavor profiles. Supplementation with 0.15% SA extract optimally enhanced both growth performance and muscle quality while maintaining nutritional value, supporting the potential utility of this invasive species as a sustainable aquafeed additive. This study provides novel foundations for the regulation of flavor quality in aquatic food animals.

## 1. Introduction

Aquaculture products constitute a crucial source of high-quality, cost-effective animal protein. With the improvements in living standards, market demand has undergone a notable shift toward aquatic products that offer superior quality, enhanced nutritional value, and additional health benefits. Nevertheless, intensive farming practices, marked by large-scale operations and high stocking densities, coupled with the progressive deterioration of aquatic environments have exerted a detrimental impact on the flavor quality of farmed species [1]. As a result, flavor quality improvement is a key research focus in contemporary aquaculture science.

The flavor quality of aquatic products is primarily determined by the interplay of volatile and non-volatile compounds. Volatile flavor components, predominantly aldehydes, esters, and alcohols, contribute aromatic notes ranging from marine to sweet and fruity profiles [2,3]. Meanwhile, non-volatile constituents, including free amino acids, nucleotides, and organic acids, collectively create the fundamental taste spectrum encompassing umami, sweetness, bitterness, and saltiness [4,5]. In addressing flavor quality challenges, research highlights the considerable potential of phytogenic feed additives. Plant-derived extracts are particularly advantageous owing to their rich bioactive compounds, nutritional benefits, and safety profiles (being residue-free and environmentally friendly). Empirical evidence demonstrates their efficacy in improving flavor profiles, muscle quality, and growth performance across various species. For instance, garlic extract (6 g/L) significantly reduces odorous compounds relative to controls [6], while traditional Chinese herbs, such as *Astragalus membranaceus* and *Angelica sinensis*, elevate umami substance concentrations in *Oreochromis niloticus* muscle while optimizing flavor characteristics [7].

*Spartina alterniflora* (SA), indigenous to the Atlantic coast of the United States, was introduced to China in 1979. It has since become the most detrimental invasive plant in coastal wetlands and is listed among China’s top 16 invasive alien species by the National Environmental Protection Agency [8]. This halophytic plant primarily contains several major classes of bioactive components, including saccharides, amino acids, proteins, flavonoids, organic acids, coumarins, and alkaloids. Among these, flavonoids are particularly abundant, and are associated with diverse pharmacological properties such as anti-inflammatory, hypoglycemic, hypolipidemic, and immunomodulatory effects [9]. Emerging research highlights the potential applications of SA in aquaculture. For instance, biomineral solutions derived from SA have been shown to enhance growth rates in several commercially important species, including grass carp (*Ctenopharyngodon idellus*), tilapia (*Oreochromis niloticus*), and blunt snout bream (*Megalobrama amblycephala*). Further, it has been demonstrated that SA biomineral solutions not only improve growth performance but also significantly enhance meat quality in *Monopterus albus* [10]. Despite these findings, the specific effects of SA on these flavor-related compounds in aquatic species remain underexplored.

The mud crab, a premium marine food animal, is widely distributed across tropical regions of the western Pacific, with substantial populations thriving along China’s southeastern coastal areas. Renowned for its delicate flavor, outstanding nutritional composition, and medicinal value, it has been conferred the accolade of “marine ginseng” [11]. The present study investigates the effects of SA extract on the growth performance and flavor quality of mud crabs, while concurrently exploring strategies for the sustainable utilization of SA and contributing to the development of innovative feed additives.

## 2. Materials and Methods

### 2.1. Diet Formulation

The experimental diets were formulated using fish meal and soybean meal as primary protein sources, supplemented with fish oil and soybean oil as lipid sources. SA extract provided by Prof. Pei Qin from Nanjing University, in collaboration with Jiangsu Shibeitai Biotech Co. (Nanjing, Jiangsu, China), was incorporated at four graded levels (0.05%, 0.1%, 0.15%, and 0.2%; see Table S1 for complete formulation), while other ingredients were of commercial grade. All dry ingredients were ground to pass through an 80-mesh sieve and homogenized using a V-type stainless steel mixer. Lipid components were sequentially added as follows: (1) fish oil/soybean oil blend (1:1 ratio); (2) yeast extract; and (3) distilled water (20% of total weight) to achieve proper pellet binding. The mixture was extruded through a 1.0 mm die using a twin-screw extruder (Model F-26, Guangzhou Huazhong Optoelectronic Technology, Guangzhou, China) and dried at 60 °C for 8 h to ≤10% moisture content. Final products were vacuum-sealed and stored at −20 °C until use. Proximate composition was determined according to AOAC (2003) methods: crude protein via Kjeldahl (N × 6.25) and crude lipid by Soxhlet extraction with petroleum ether [12].

### 2.2. Spartina Alterniflora (SA) Extract

SA extract is classified as a New Resource Food (Wei-Xin-Shi-Zhun-Zi-94-N.06) developed and produced by The Research Team on *Spartina*, Nanjing University and Jiangsu Shibeitai Biotech Co. Extensive studies in both animals and humans indicate that the extracts have significant immunity-improving effects. The SA extract used in this study, namely Spartina Powder, is a yellowish powder with excellent water solubility and a savory taste. Its primary nutritional components include polysaccharides (≥300 mg/g), saponins (≥50 mg/g), and flavonoids (≥30 mg/g) [13].

### 2.3. Animals and Feeding Trial

Juvenile mud crabs were sourced from Xianyin Aquaculture Farm (Sanmen County, Ningbo, China) and transported to the experimental facility at Xuwen Seaweed Development Co., Ltd. (Xiangshan County, Ningbo, China). Following a 7-day acclimation period in concrete rearing tanks (16 m × 12 m × 1 m), 150 healthy crabs of uniform size (mean initial weight 21.85 ± 3.54 g) were selected and randomly distributed into 5 experimental groups (3 replicates per group, 10 crabs per replicate). To minimize cannibalism, each crab was individually maintained in a partitioned culture box (20 cm × 40 cm × 30 cm) within the main tanks. The 9-week feeding trial was conducted under controlled conditions, with crabs fed twice daily (08:00 and 17:00) at 4% of their body weight. Residual feed and feces were removed daily, and dead or molted crabs were promptly recorded and removed. Additionally, one-third of the total water volume was replaced every 2 days. Throughout the trial, the sea water quality parameters were maintained as follows: dissolved oxygen > 5 mg/L, ammonia nitrogen < 0.2 mg/L, temperature 27–30 °C, and pH 8.1–9.0.

### 2.4. Sample Collection

During the 9-week feeding trial, all surviving crabs molted at least twice, indicating normal physiological development. Upon termination of the experiment, after a standardized 24 h fasting period to ensure gut clearance, 4 crabs were randomly selected from each replicate tank (each treatment comprising 3 replicate tanks, totaling 12 crabs per treatment group). Each specimen was carefully blotted dry with absorbent paper before the following morphological measurements were taken: body weight (measured to the nearest 0.01 g using an analytical balance) and carapace dimensions (length and width recorded with digital vernier calipers). Subsequently, muscle tissue samples were aseptically excised from the cheliped using sterilized forceps, with tissues from the 4 crabs pooled to form one composite sample per replicate. All samples were flash-frozen in liquid nitrogen and subsequently stored at −80 °C until biochemical analysis. Growth performance was evaluated using 3 key metrics:

Weight gain rate (WGR, %) = (final weight − initial weight)/initial weight × 100

Condition factor (CF, %)=body weight/length^3^ × 100

Specific growth rate (SGR, % d^−1^) = 100 × (Ln Wf − Ln Wi)/t

where Wf refers to the body weight after time t, Wi represents the initial body weight, and t is the number of breeding days.

### 2.5. Electronic Tongue

The taste profile analysis was conducted using an electronic tongue system (Insent Taste Sensing System, Insent Inc., Takarazuka, Japan) following an adapted version of the Buratti method [14]. The system quantitatively assessed 6 primary taste attributes (umami, saltiness, sourness, bitterness, astringency, and richness) along with 2 aftertaste components (bitter aftertaste and astringent aftertaste). For sample preparation, crab meat samples (2.00 ± 0.01 g) were homogenized in 25 mL ultrapure water using a sterile blender. The homogenate underwent sequential processing: (1) ultrasonic extraction (5 min at 40 kHz); (2) room temperature incubation (30 min); and (3) centrifugation (12,000× *g*, 15 min, 4 °C). The resulting supernatant was filtered through 0.45 μm membrane filters, with the pellet subjected to a second extraction under identical conditions. The combined filtrates were brought to 100 mL with ultrapure water, and 5 mL aliquots were diluted to 80 mL for analysis (25 ± 1 °C). The electronic tongue operation protocol consisted of 4 standardized steps: First, sensor electrodes were equilibrated in a tartaric acid-modified KCl reference solution (Vr). Subsequently, sample measurements (Vs) were performed to determine initial taste intensity (ΔV = Vs − Vr). Following a 6 s automated rinse cycle, reference potential was re-measured (Vr’) to calculate aftertaste intensity (ΔV’ = Vr’ − Vr). Between samples, electrodes underwent three cleaning cycles with ultrapure water to prevent cross-contamination. All measurements were conducted in triplicate, with system calibration performed every 10 samples using standard reference solutions.

### 2.6. Free Amino Acids

Free amino acid quantification was performed using an optimized method adapted from Luo et al. [15]. Briefly, 0.4 g of lyophilized crustacean muscle tissue was weighed into a 50 mL centrifuge tube and homogenized with 10 mL of chilled 4% (*w*/*v*) sulfosalicylic acid solution. To prevent thermal degradation, ultrasonic extraction was carried out in an ice-water bath for 60 min, followed by refrigeration at 4 °C for 2 h to facilitate protein precipitation. After centrifugation (5000× *g*, 10 min, 4 °C), the supernatant was collected in a volumetric flask. The extraction was repeated with fresh solvent, and the combined extracts were adjusted to 25 mL with ultrapure water. Following vigorous vortexing (30 s) to ensure homogeneity, the samples were filtered (0.22 μm aqueous filter) into HPLC vials and stored at −20 °C until derivatization (within 48 h).

For flavor compound analysis, taste activity values (TAVs) were calculated using the equation: TAV = C/T, where C represents compound concentration and T denotes the detection threshold. Compounds with TAVs > 1 were considered sensorially significant.

### 2.7. Flavor Nucleotides

Nucleotide content analysis was performed using a modified method based on Yu et al. [16]. Briefly, 5.00 g of muscle tissue samples was homogenized with 25 mL of 0.6 M perchloric acid for 2 min, followed by continuous shaking for 20 min. After centrifugation (13,500× *g*, 10 min), the supernatant was adjusted to pH 7.0 with 1.0 M NaOH and recentrifuged under identical conditions. The neutralized extract was then diluted to 50 mL, filtered (0.45 μm), and analyzed by HPLC. Separation was achieved using a Diamonsil C18 column (250 × 4.6 mm) maintained at 30 °C with a flow rate of 0.8 mL/min. The mobile phase consisted of two solvents, (A) methanol-water-H_3_PO_4_ (5:95:0.05, *v*/*v*) and (B) methanol-water-H_3_PO_4_ (80:20:0.05, *v*/*v*), with UV detection at 254 nm.

The equivalent umami concentration (EUC), expressed as grams of monosodium glutamate (MSG) per 100 g sample, was calculated to evaluate the synergistic umami intensity between taste-active components, using the established formula: EUC = Σ(AᵢBᵢ) + 1218[Σ(AᵢBᵢ)][Σ(AⱼBⱼ)], where Aᵢ and Aⱼ represent the concentrations (g/100 g) of umami amino acids (aspartic acid [Asp] and glutamic acid [Glu]) and 5′-ribonucleotides (AMP, GMP, IMP), respectively; Bᵢ and Bⱼ denote their relative umami contribution factors (Asp = 0.077, Glu = 1.000; AMP = 0.18, GMP = 2.3, IMP = 0.18); and 1218 is the empirically derived synergistic constant.

### 2.8. Fatty Acids

An accurately weighed sample was homogenized in chloroform/methanol/BHT (2:1:0.01) and stored at 4 °C overnight. The mixture was subsequently filtered, and calcium chloride solution was added to the filtrate with thorough vortexing. After centrifugation, the supernatant was discarded, followed by the addition of a desiccant and standing at room temperature. The solution was filtered again, and the filtrate was evaporated under nitrogen stream. Internal standard and toluene were added to the residue with nitrogen purging and vigorous vortexing, followed by the addition of sodium methoxide and incubation in a 60 °C water bath. Boron trifluoride/methanol solution was then introduced with nitrogen purging and thorough vortexing. After another 60 °C water-bath treatment, the mixture was rapidly cooled to room temperature and treated with sodium bicarbonate solution. Following standing, n-hexane was added, with vigorous shaking and subsequent phase separation. A desiccant was incorporated, and the mixture was kept at room temperature before final filtration. The resulting filtrate was concentrated under nitrogen and reconstituted in n-hexane for analysis. The GC conditions were as follows: (a) CP-Sil 88 capillary column (100 m × 0.25 mm × 0.2 μm) specifically designed for fatty acid methyl ester analysis; (b) injector temperature maintained at 280 °C; (c) FID detector operated at 300 °C with temperature programming: initial temperature 130 °C (hold 5 min), ramped at 4 °C/min to 240 °C (hold 20 min), yielding a total run time of 52.5 min; (d) carrier gas: high-purity nitrogen (99.99%); and (e) injection volume: 1 μL.

### 2.9. Electronic Nose

The volatile profile of crab meat was analyzed using a PEN3 electronic nose (Airsense Analytics, Schwerin, Germany). For each measurement, 2.00 ± 0.01 g of sample was precisely weighed into a 10 mL headspace vial, which was then sealed with parafilm and equilibrated at 60 °C for 15 min to allow volatile compound accumulation. The instrument parameters were set as follows: 120 s purge and measurement phases, 1 s sampling interval, and 5 s preparation time, with both inlet and sampling flow rates maintained at 400 mL/min.

### 2.10. Volatile Compounds

Volatile compounds in crab meat were analyzed using headspace solid-phase microextraction (HS-SPME) coupled with GC-MS. Precisely 2.0 g of sample was weighed into a 20 mL headspace vial. A preconditioned SPME fiber was inserted through the septum and exposed to the headspace for 40 min at 100 °C. After extraction, the fiber was desorbed in the GC-MS injector at 240 °C for 5 min.

GC-MS analysis was conducted in triplicate on a DB-5MS column (60 m × 0.32 mm, 1 μm film thickness) with helium as the carrier gas (1.0 mL/min, splitless mode). The oven temperature program was as follows: initial temperature of 40 °C, ramped to 100 °C at 5 °C/min, then to 180 °C at 3 °C/min, and finally to 240 °C at 5 °C/min (held for 5 min). The injector temperature was maintained at 240 °C throughout the analysis.

### 2.11. Statistics, Calculations, and Visualization Analysis

According to the principle of parallelism, all experiments were performed at least three times (*n* = 3). Statistical analyses were conducted using SPSS 20.0 (IBM Corp., Armonk, NY, USA). Prior to parametric testing, assumptions of homogeneity of variance (Levene’s test) and normal distribution (Shapiro–Wilk test) were verified. Significant differences among groups (*p* < 0.05) were determined by one-way ANOVA followed by Duncan’s multiple range test. Results are presented as mean ± standard error of the mean (SEM). Intervariable relationships were assessed using Pearson’s correlation analysis, with statistical significance set at *p* < 0.05 and meaningful correlations defined as |r| > 0.50. Data visualization was performed using specialized software. All bar graphs were generated with GraphPad Prism 10. Principal component analysis (PCA) plots and radar charts were created using Origin 2021. Heatmaps and comprehensive correlation analysis plots were produced with online platforms: the former using the bioinformatics online platform, and the latter employing the Chiplot 2025 platform.

## 3. Results and Discussion

### 3.1. Effects of Different Concentrations of SA Extract on Growth Performance of Mud Crab

**Figure 1:**
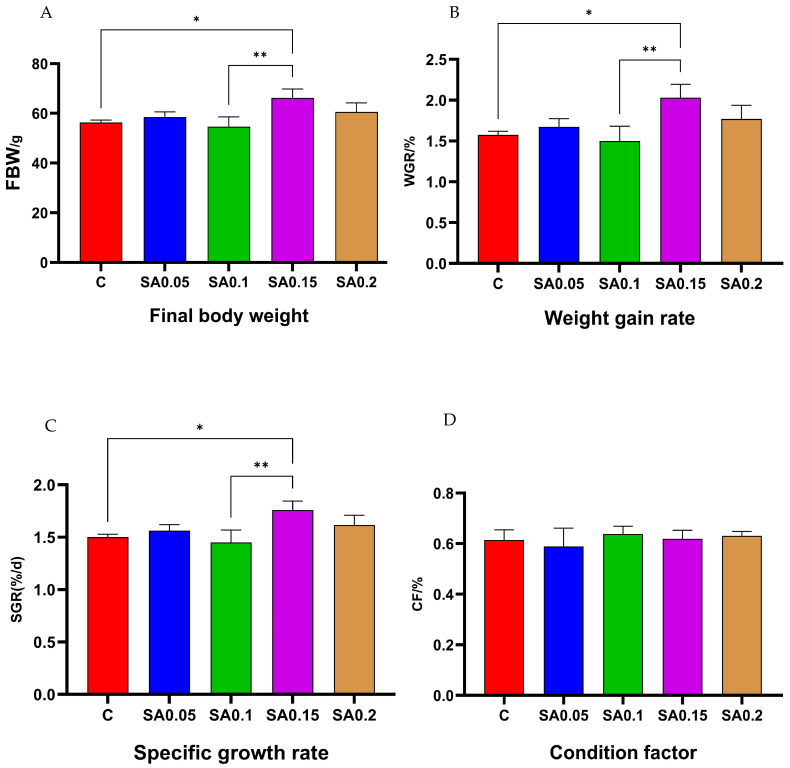
Effects of different concentrations of SA extract on growth indexes of mud crab (*n* = 3), (**A**) Body weight; (**B**) Weight gain rate; (**C**) Specific growth rate; (**D**) Condition factor. Stars represent statistically significant differences at *p* < 0.05 (*) or *p* < 0.01(**).

As shown in Figure 1, the growth performance of mud crabs varied significantly between the dietary treatments. Compared with the CG, crabs fed with 0.05%, 0.15%, and 0.2% SA extract supplements showed increased final body weight (FBW), weight gain rate (WGR), and specific growth rate (SGR). These increases were particularly observed in the 0.15% group, where these parameters were significantly higher (*p* < 0.05). These effects may be attributed to the bioactive compounds present in SA extract, including polysaccharides (known to enhance feed efficiency and nutrient absorption), saponins, and flavonoids. Supporting this observation, Wang et al. [17] demonstrated that similar plant-derived compounds, such as mulberry leaf flavonoids and polysaccharides, significantly boosted growth performance in largemouth bass (*Micropterus salmoides*). The Complexed Oral Liquid of Spartina (Wei-Shi-Jian-Zi-1997-N.321), prepared using Spartina extracts as raw material, increases the phagocytosis of macrophages and strengthens nonspecific immunity. Further animal and human tests have shown that Spartina extracts reduce uric acid levels and confer significant protective effects against gout [13]. Notably, the 0.1% supplementation group exhibited a marginal, statistically non-significant (*p* > 0.05) growth reduction. This phenomenon may have stemmed from either intrinsic growth rate variability among individual crabs or transient confounding factors (e.g., feeding activity fluctuations, molting phase synchronization, or microenvironmental variations), rather than reflecting genuine biological responses to SA extract. No significant differences (*p* > 0.05) were detected in the CF between the CG and all treatment groups. Although significant growth enhancement was observed at 0.15% and 0.2% supplementation levels, the consistent CF values across groups indicate that SA extract specifically promoted body weight gain without body conformation.

### 3.2. Effects of Different Concentrations of SA Extract on Electronic Tongue Parameters of Mud Crab

**Figure 2:**
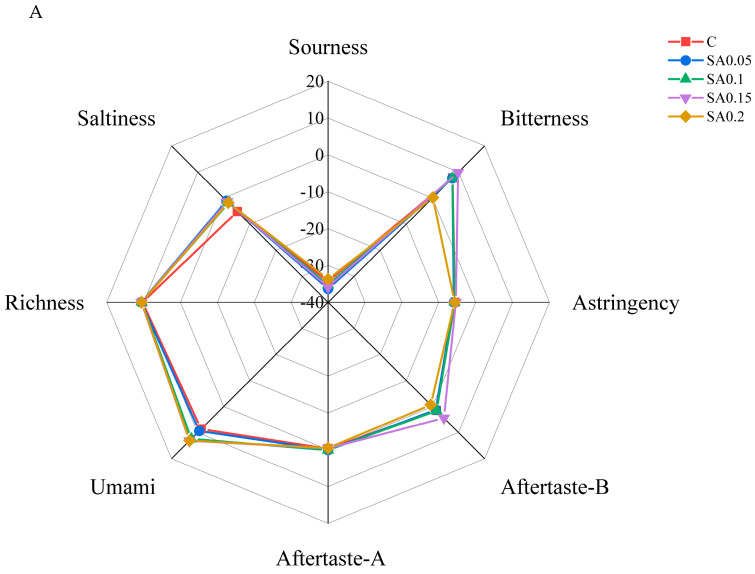

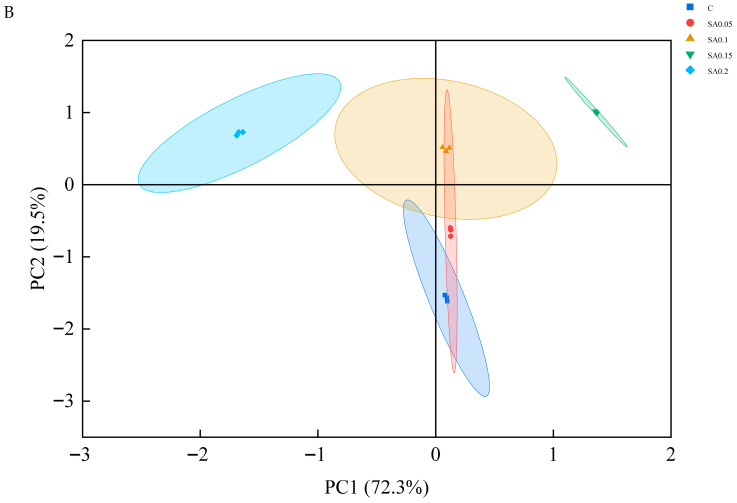
Electronic tongue radar plot (**A**) and principal component analysis (**B**) of crab flesh.

The electronic tongue radar plot (Figure 2A) revealed distinct differences between the groups, particularly in bitterness, bitter aftertaste, umami, and saltiness. Specifically, the 0.2% group exhibited lower bitterness and bitter aftertaste intensity values than the CG, while the umami intensity of the 0.1%, 0.15%, and 0.2% groups was significantly higher than that of the control. Additionally, the CG displayed lower saltiness than the other groups.

To further analyze the taste differences, principal component analysis (PCA) was employed, given its proven effectiveness in extracting key information from electronic tongue data [18]. The results showed that the cumulative contribution rate of the first two principal components reached 91.8%, exceeding the 85% threshold, indicating minimal loss of variability in the flavor profile of mud crab muscle. Notably, the CG clustered closely with the 0.05% and 0.1% supplementation groups, suggesting that dietary incorporation of 0.05–0.1% SA extract exerted minimal effects on the taste characteristics of crab meat. In contrast, distinct separation was observed between the CG and both the 0.15% and 0.2% treatment groups, demonstrating that higher supplementation levels (0.15–0.2%) of SA extract significantly altered the taste profile of crab meat.

### 3.3. Effects of Different Concentrations of SA Extract on Free Amino Acids of Mud Crab

**Figure 3:**
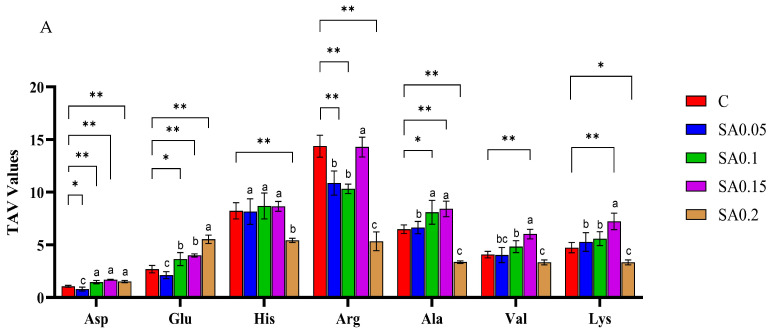

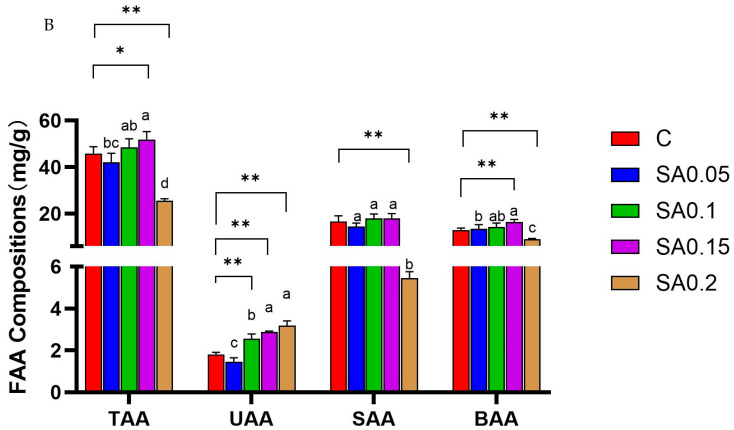
Effect of SA extract on free amino acids of mud crab. (**A**) taste free amino acid content of crab flesh (mg/g); (**B**) TAV values of free amino acids of crab flesh. Values are expressed as means ± SD (*n* = 3). Difference from C: * = *p* < 0.05, ** = *p* < 0.01. Different superscripts denote significant differences between treatments (*p* < 0.05).

Taste constitutes a key factor influencing consumers’ purchasing decisions. Free amino acids play a dual role in flavor perception, directly contributing to taste while also participating in the formation of flavor compounds. Moreover, synergistic interactions among different amino acids can further enhance palatability, resulting in a more desirable taste profile [19,20]. As illustrated in Table 1 and Figure 3A, 17 amino acids were detected in mud crab muscle (Table 1), with arginine (Arg) being the most abundant, followed by glycine (Gly) and proline (Pro)—a composition consistent with previous findings in snow crab leg meat [21]. All treatment groups (0.1%, 0.15%, and 0.2%) exhibited significantly elevated umami-associated amino acid content compared with the CG (*p* < 0.01). Notably, while the 0.15% group showed the highest bitter-tasting amino acid concentration among all groups (*p* < 0.05 versus CG), this trend was reversed in the 0.2% group, which demonstrated significantly reduced bitter-tasting amino acids (*p* < 0.05). These findings were corroborated by electronic tongue analysis, confirming the taste profile modifications. Regarding total amino acid content, both 0.1% and 0.15% supplementation groups surpassed the CG, with the 0.15% group reaching statistical significance (*p*< 0.05). In contrast, the 0.2% group displayed a distinct pattern, showing significantly lower levels of both total amino acids and sweet-tasting amino acids relative to other treatment groups (*p* < 0.05). This suggests an optimal supplementation range between 0.15% and 0.2% for balancing flavor enhancement and amino acid preservation. The findings reveal that dietary supplementation with 0.1–0.2% SA extract significantly enhanced the total free amino acid content in mud crab muscle. Notably, supplementation levels ≥ 0.1% induced a marked increase in umami-associated amino acids (*p* < 0.05), thereby improving the overall flavor profile of crab meat. While the 0.2% group showed the greatest umami amino acid enhancement, it also demonstrated significantly lower total amino acid content than the other groups (*p* < 0.05). SAE contains abundant flavonoids that have been shown to regulate glycolytic enzyme synthesis in mouse liver [22]. This suggests that flavonoids in SAE may similarly modulate the synthesis of key enzymes involved in amino acid metabolism in crab muscle, particularly promoting the production of umami-associated amino acids. These observations align with previous reports by Zhou and Leng regarding flavonoid-rich plant additives [23,24]. Notably, while the 0.2% SAE group exhibited the most pronounced increase in umami amino acids, it showed significantly lower total amino acid content than other groups (*p* < 0.05). This apparent paradox may be explained by concentration-dependent effects of flavonoids: at higher concentrations (0.2%), these compounds might preferentially upregulate enzymes specific to umami amino acid synthesis pathways while simultaneously inhibiting other amino acid metabolic pathways or enhancing amino acid catabolism. Alternatively, the flavonoids could be redirecting metabolic flux toward umami amino acid production at the expense of other amino acids.

The taste activity value (TAV), defined as the ratio of the concentration of a taste-active substance to its taste threshold, serves as a key indicator of its flavor contribution. A TAV ≥ 1 signifies that the substance significantly influences the overall taste profile, with higher values indicating greater contribution [25]. Our analysis identified 7 key taste-active amino acids (Figure 3B). With the exception of Asp in the 0.05% group (TAV < 1), all examined free amino acids (Arg, His, Ala, Lys, Val, Glu, and Asp) exhibited TAV values ≥ 1 across treatment groups, directly impacting crab muscle flavor characteristics. Notably, the 0.1%, 0.15%, and 0.2% groups showed significantly higher TAV values for Asp and Glu than the CG (*p* < 0.05). As the primary umami amino acids, Asp and Glu synergize with flavor nucleotides to enhance umami perception—a key factor contributing to the distinctive, high-value flavor profile of mud crab [26]. Further analysis revealed that the 0.15% and 0.1% groups exhibited significantly higher TAV values for Ala compared with the control group (*p* < 0.01). As a sweet-tasting amino acid with subtle bitter undertones, Ala demonstrates pronounced flavor-enhancing synergy when combined with umami compounds such as glutamate and inosine monophosphate. Notably, the 0.15% group showed significantly elevated TAV values for 2 bitter-tasting amino acids Lys and Val relative to all other groups (*p* < 0.05), which corresponded to its stronger bitter profile in electronic nose analysis. In contrast, the 0.2% supplementation group displayed a fundamentally different pattern, with significantly reduced TAVs for 7 free amino acids (*p* < 0.05). Most notably, the decreased levels of bitter-tasting amino acids—particularly histidine (His), Lys, and Val—were primarily responsible for the substantially weaker bitter attributes detected in this group. These findings suggest a concentration threshold effect, where higher SA extract supplementation (≥0.2%) may selectively suppress certain amino acid metabolic pathways while preserving umami characteristics.

### 3.4. Effects of Different Concentrations of SA Extract on Flavor Nucleotides of Mud Crab

**Figure 4:**
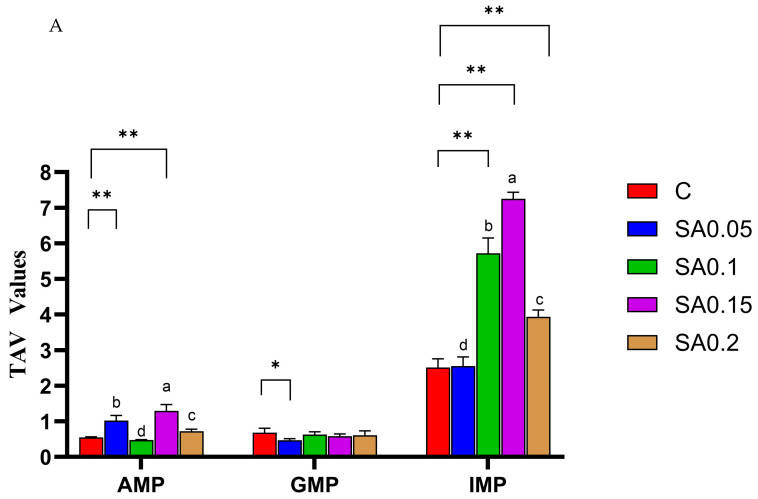

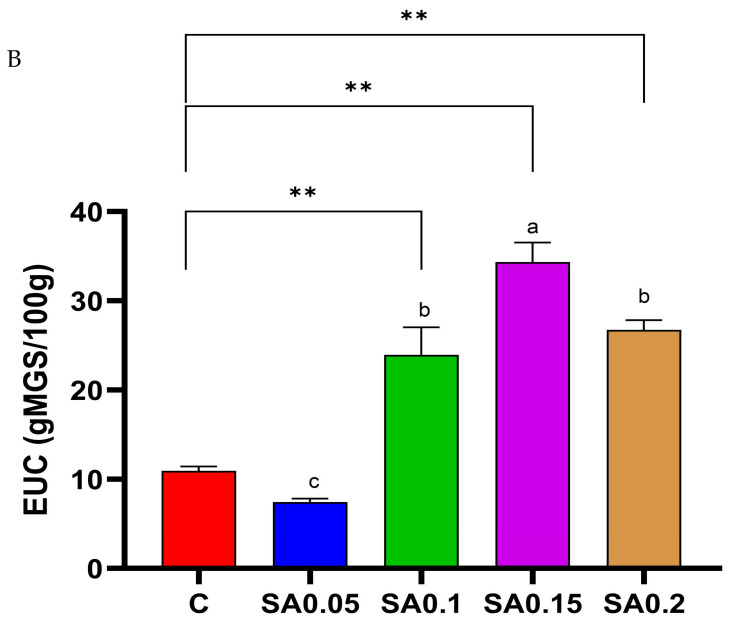
Effect of SA extract on nucleotides of mud crab. (**A**) TAV values of crab flesh; (**B**) EUC (g MSG/100 g) of crab flesh. Values are expressed as means ± SD (*n* = 3). Difference from C: * = *p* < 0.05, ** = *p* < 0.01. Different superscripts denote significant differences between treatments (*p* < 0.05).

Beyond free amino acids, nucleotides constitute another crucial group of flavor-active compounds, with their umami characteristics being particularly significant [15]. Our results demonstrated that inosine monophosphate (IMP) was the predominant nucleotide in mud crab muscle, exhibiting higher concentrations than adenosine monophosphate (AMP) and guanosine monophosphate (GMP) (Table 2). This distribution pattern agrees with previous reports by Wang et al. and Shi et al. [27,28]. The 0.15% supplementation group exhibited significantly higher concentrations of both AMP and IMP compared with other treatment groups (*p* < 0.05). In contrast, the CG showed significantly lower IMP levels than the 0.1%, 0.15%, and 0.2% supplementation groups (*p* < 0.05). However, its GMP content was only significantly elevated relative to the 0.05% group, with no statistically significant differences observed between the other groups. As a crucial flavor precursor, IMP contributes to flavor development by providing ribose for Maillard reactions [29]. Notably, Figure 4A demonstrates that among all detected nucleotides, only IMP displayed taste activity values (TAVs) >1 (the flavor contribution threshold) across all treatment groups, further confirming its predominant role in mud crab flavor formation. Specifically, the 0.1%, 0.15%, and 0.2% supplementation groups exhibited significantly elevated IMP TAV values relative to the control group. These findings corroborate Zhou et al.’s report [23] that mulberry leaf powder—containing bioactive components analogous to SA extract—effectively enhances IMP levels in fish muscle.

While TAV is an effective index to evaluate individual flavor compounds’ contributions, it fails to account for potential masking or synergistic effects among multiple flavor substances [30]. The equivalent umami concentration (EUC) addresses this limitation by quantitatively assessing the synergistic enhancement between umami amino acids (Asp and Glu) and nucleotides (AMP, GMP, and IMP), providing a more accurate characterization of umami intensity in complex systems [31]. Our results revealed that the 0.1%, 0.15%, and 0.2% treatment groups showed significantly higher EUC values than the control (*p* < 0.01) (Figure 4B), indicating that SA extract supplementation effectively enhanced mud crab muscle’s umami characteristics. Notably, the 0.15% addition level demonstrated the most pronounced effect, suggesting an optimal concentration range for umami enhancement.

### 3.5. Effects of Different Concentrations of SA Extract on Fatty Acid Composition of Mud Crab

**Figure 5:**
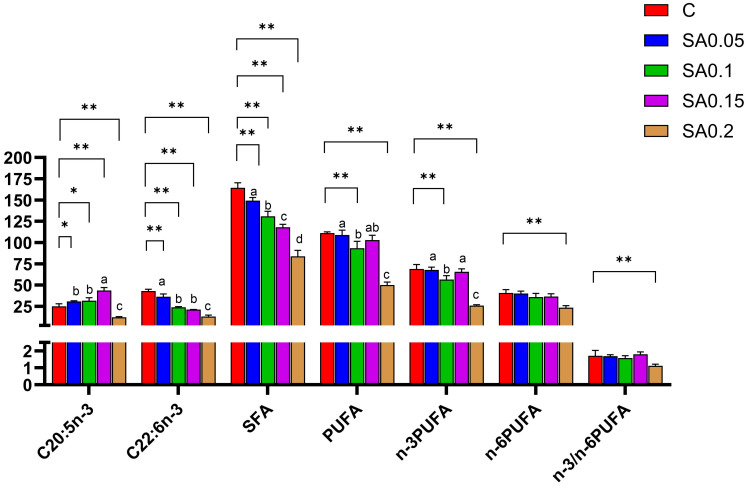
Effect of SA extract on fatty acids of mud crab. Main fatty acid content in mud crab flesh. Values are expressed as means ± SD (*n* = 3). Difference from C: * = *p* < 0.05, ** = *p* < 0.01. Different superscripts denote significant differences between treatments (*p* < 0.05).

As shown in Table 3 and Figure 5, dietary supplementation with *S. alterniflora* extract significantly altered the fatty acid profile of mud crabs. A total of 37 fatty acids were identified, with saturated fatty acids (SFAs), primarily palmitic acid (C16:0) and tetracosanoic acid (C24:0), being the most abundant. The SFA content in the control group was significantly higher than in all treatment groups (*p* < 0.01), showing a dose-dependent decrease with increasing SA extract concentrations. This reduction may result from the inhibitory effects of bioactive compounds in the extract, particularly flavonoids, on SFA biosynthesis [32]. Further analysis indicated that palmitic acid levels in the CG were significantly higher than in other groups (*p* < 0.05), whereas tetracosanoic acid levels were elevated in both the CG and the 0.05% supplementation group relative to higher-dose treatments. Notably, excessive dietary SFA intake is strongly linked to cardiovascular disease risk. Palmitic acid, as a predominant dietary SFA, promotes arterial cholesterol deposition, thereby increasing atherosclerosis risk [33]. These findings suggest that SA extract supplementation can modulate SFA levels in mud crabs, potentially enhancing their nutritional value by mitigating health risks associated with high SFA consumption.

Polyunsaturated fatty acids (PUFAs), primarily docosahexaenoic acid (DHA, C22:6n-3) and eicosapentaenoic acid (EPA, C20:5n-3), represented the second most abundant fatty acid class. Among the experimental groups, the control group exhibited significantly higher total PUFA content than all treatment groups (*p* < 0.05), particularly compared with the 0.1% and 0.2% supplementation groups. In contrast, the 0.2% treatment group showed the lowest PUFA levels across all groups. Regarding individual fatty acids, EPA levels were significantly higher in the 0.05%, 0.1%, and 0.15% treatment groups than in the control (*p* < 0.05), while the control group displayed markedly elevated DHA content (*p* < 0.01). PUFAs provide multiple health benefits, e.g., EPA and DHA support neurodevelopment and regulate lipid metabolism [34]. Additionally, PUFAs can enhance meat flavor through oxidation-derived compounds [35]. The n-3/n-6 PUFA ratio serves as a critical indicator for assessing both nutritional quality and flavor characteristics. According to Food and Agriculture Organization (FAO) recommendations, the optimal n-3/n-6 PUFA ratio in food should exceed 0.1–0.2 [36,37]. Although SA extract reduced the DHA content in mud crab muscle, EPA levels significantly increased in all groups except the 0.2% group, while the n-3/n-6 ratio remained stable. These findings align with those of Li et al. [38], who reported that EPA improves muscle quality in grass carp by upregulating mTOR signaling pathway-related genes and proteins. This mechanism may explain the optimal growth performance observed in our 0.15% group, which exhibited the highest EPA content. Notably, the 0.2% treatment group showed significantly lower levels of all major fatty acids compared with the control group (*p* < 0.01; Figure 5), suggesting that excessive SA extract supplementation may inhibit fatty acid synthesis and/or enhance catabolism. Collectively, these results demonstrate that supplementing feed with appropriate concentrations of SA extract (≤0.15%) optimizes fatty acid composition, thereby improving the flavor-nutrition balance in crab muscle.

### 3.6. Effects of Different Concentrations of SA Extract on Electronic Nose Analysis Results of Mud Crab

**Figure 6:**
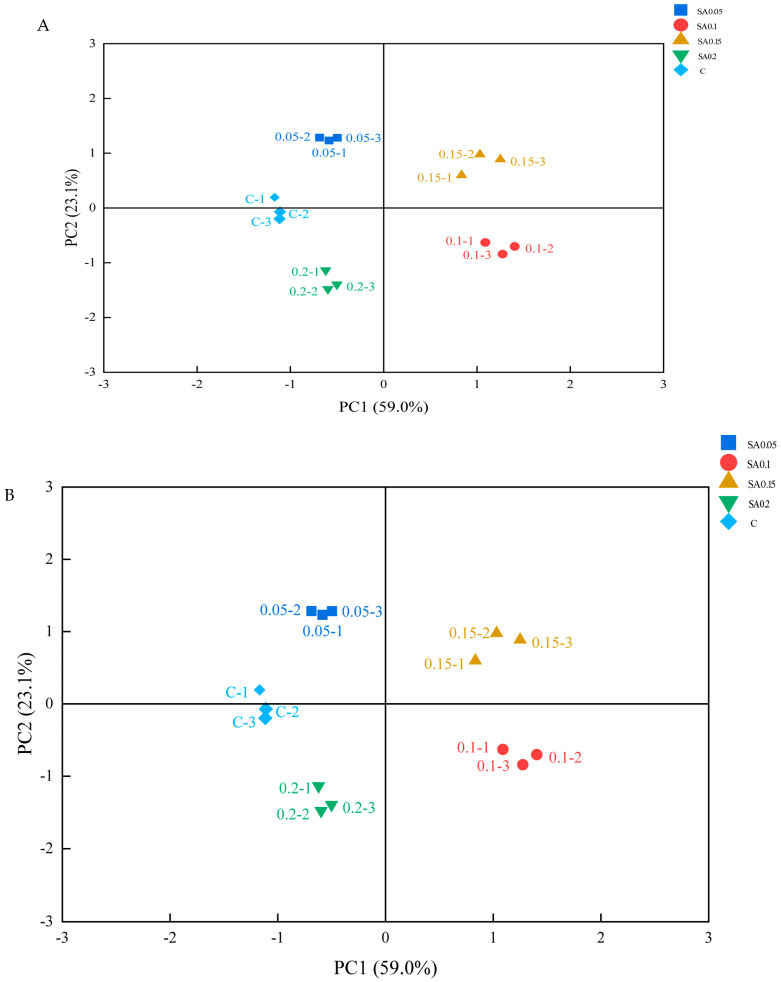

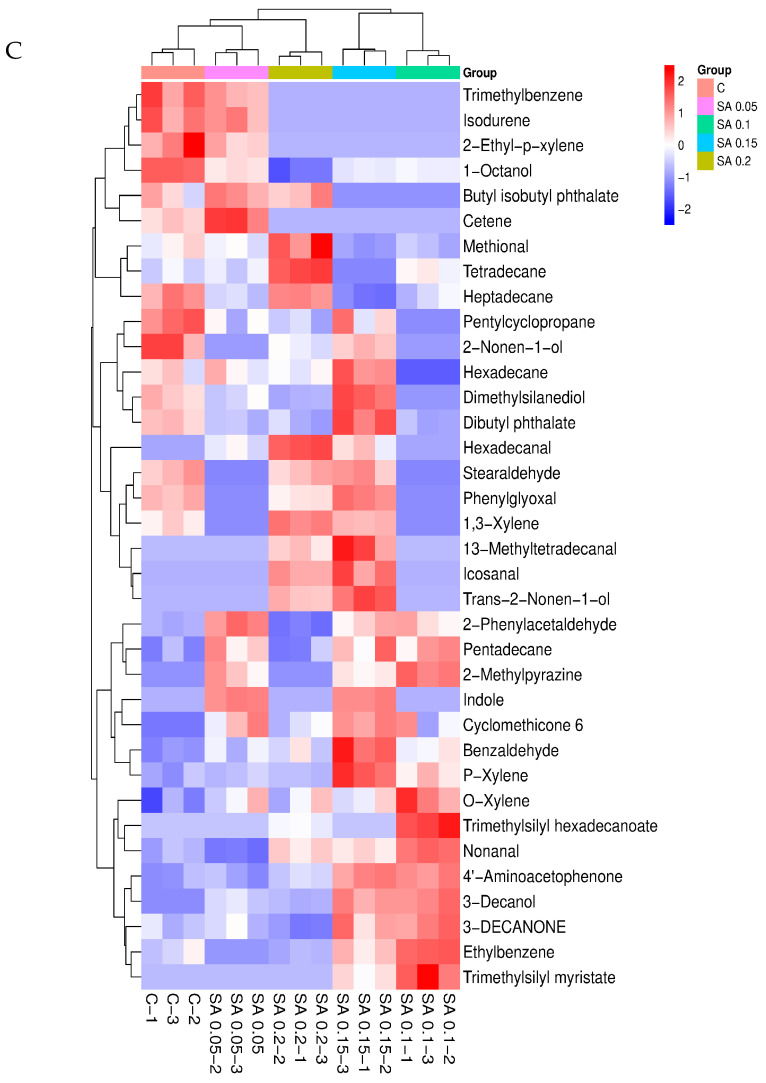
Effect of SA extract on volatile compounds present in mud crab flesh. (**A**) Principal component analysis (PCA) of all volatile compounds. (**B**) Principal component analysis (PCA) of electronic nose data. (**C**) Hierarchical cluster analysis (HCA) and heat map visualization of samples and volatile compounds in crab flesh.

PCA of electronic nose data (Figure 6B) showed that the first two principal components (PC1 and PC2) explained 97.7% of the total variance, exceeding the 95% threshold required for effective odor characterization. Clear clustering patterns with no overlap between treatment groups indicated that SA extract supplementation significantly altered the volatile compound profile of crab meat. The most pronounced difference was observed between the CG and both the 0.1% and 0.15% treatment groups, suggesting that these concentrations had the greatest impact on the volatile compound profile of crab meat. Although the CG also showed distinct distributions from the 0.05% and 0.2% groups, these differences were primarily along PC2, which accounted for only 22.9% of total variance. This indicates that differences between the control and 0.1%/0.15% groups were more significant than those between the control and 0.05%/0.2% groups.

### 3.7. Effects of Different Concentrations of SA Extract on Volatile Compound of Mud Crab

GC-MS analysis identified 36 volatile compounds in mud crab muscle (Table 4), aligning with reported profiles for mud crabs and swimming crabs (*Portunus* spp.) [15,39]. The composition primarily included 9 aldehydes, 4 alcohols, 2 ketones, 4 esters, 8 hydrocarbons, 7 aromatic compounds, and 2 other compounds. Aldehydes dominated the volatile profile, consistent with their established role as key flavor contributors owing to both their high abundance and low odor thresholds [39]. In the present study, aldehydes constituted the predominant class of volatile compounds in crab muscle, with nonanal, benzaldehyde, 2-phenylethanal, and 3-methylthio propanal being the most abundant. The aldehyde content in CG was significantly lower than in the other groups (*p* < 0.05). Nonanal contributes a robust flavor profile, characterized by pronounced meaty and fresh grassy notes [40], whereas benzaldehyde imparts distinct fruity and nutty aromas [41]. The concentrations of nonanal and benzaldehyde in the 0.1%, 0.15%, and 0.2% treatment groups were significantly higher than those in the CG (*p* < 0.05). Notably, the CG exhibited 6 detectable aldehyde compounds, whereas the 0.05%, 0.1%, 0.15%, and 0.2% treatment groups contained 5, 4, 9, and 9 aldehydes, respectively. Of these, icosanal and 13-methyltetradecanal were uniquely identified in the 0.15% and 0.2% groups. These two groups displayed the most diverse aldehyde profiles, with up to 9 distinct compounds detected. Although the 0.05% and 0.1% treatment groups exhibited fewer types of aldehydes compared with the CG, their total aldehyde content was significantly higher (*p* < 0.05). This suggests that dietary supplementation with SA extract at higher concentrations (≥0.15%) enhances aldehyde diversity. These aldehydes exhibit synergistic effects, generating intense flavor characteristics even at trace levels, thereby playing a crucial role in shaping the overall flavor profile of crab muscle [42].

Although alcohol compounds generally exhibit high odor perception thresholds and thus contribute minimally to overall food aroma profiles, certain unsaturated or high-concentration alcohols can still significantly influence flavor characteristics [43]. In mud crab muscle, alcohols constituted the second most abundant group of volatile compounds after aldehydes. The alcohol content in the 0.1% and 0.15% groups was significantly higher than that in the other groups (*p* < 0.05), whereas the 0.2% group showed significantly lower levels (*p* < 0.05). Major representatives of alcohol compounds included 2-nonen-1-ol, 3-decanol, 1-octen-3-ol, and trans-2-nonen-1-ol. Notably, 1-octen-3-ol—the predominant alcohol—serves as a key volatile in crustaceans, imparting characteristic mushroom, grassy, and earthy notes [44]. The CG exhibited significantly higher 1-octen-3-ol content than the experimental groups (*p* < 0.05). Notably, 4 types of alcoholic compounds were detected in both the 0.15% and 0.2% treatment groups—significantly higher than the two types identified in the CG and lower-concentration groups. These findings demonstrate that SA extract supplementation at concentrations ≥0.15% can effectively promote the generation of additional alcoholic compounds. Regarding ketones, only two compounds (4′-aminoacetophenone and 3-decanone) were detected in crab muscle. Among these, 3-decanone contributed fruity and herbal aromas [42], with its content being significantly elevated in the 0.1% and 0.15% treatment groups (*p* < 0.05). However, owing to its high odor threshold, the actual olfactory impact of 3-decanone remained limited. Our findings demonstrate that dietary SA extract supplementation exerts selective effects on volatile flavor compounds in *S. paramamosain* muscle tissue. While showing no significant influence on alcohol or ketone concentrations, SA extract effectively promoted aldehyde accumulation (*p* < 0.05). This specific modulation of aldehyde compounds, known for their low odor thresholds and significant contribution to aroma, substantially enhanced the overall flavor quality of crab meat.

PCA of the 36 identified volatile compounds in mud crab muscle tissue revealed distinct clustering patterns (Figure 6A). The first two principal components collectively explained 82.1% of total variance, with PC2 accounting for the majority (59.0%) and PC1 contributing 23.1%. While the CG and lower-concentration treatments (0.05% and 0.1%) showed minimal separation, significant compositional differences were observed in the 0.15% and 0.2% groups relative to controls. Complementary hierarchical cluster analysis (HCA) with heatmap visualization (Figure 6C) classified the treatments into two primary clusters: Cluster I (0.1% and 0.15% groups) and Cluster II (control, 0.05%, and 0.2% groups). This bipartite clustering suggests two distinct response patterns to SA extract supplementation. The integrated multivariate analysis demonstrates that 0.1–0.15% SA extract concentrations induce substantial modifications in the crab muscle volatile compound profile. Notably, the 0.2% group exhibited a divergent response, indicating a potential threshold effect or nonlinear dose–response relationship in volatile compound regulation. These findings were consistent with those from parallel electronic nose analyses, confirming the reliability of the observed patterns.

### 3.8. Correlation Analysis of Volatile Compounds, Amino Acids, and Fatty Acids in the Muscle of Mud Crabs Treated with Different Concentrations of SA Extracts

**Figure 7:**
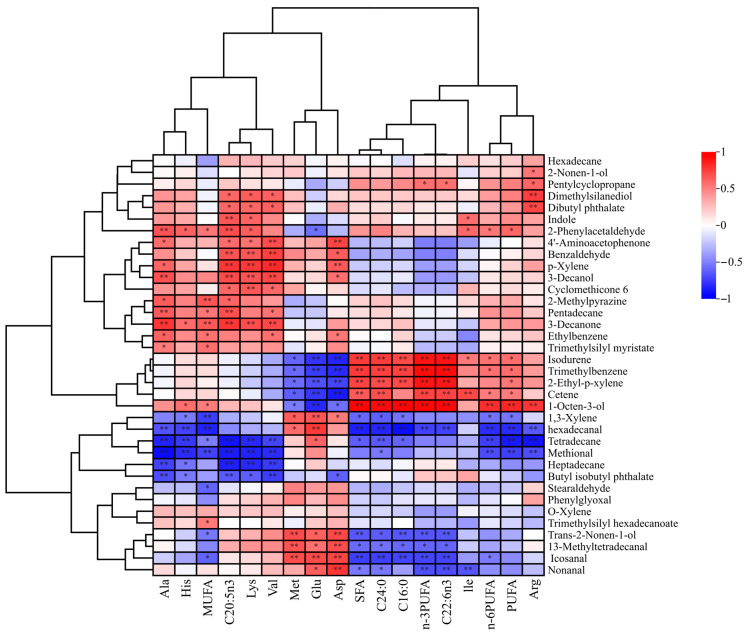
Correlation analysis of volatile compounds, amino acids and fatty acids in muscle of mud crabs treated with different concentrations of *S. alterniflora* extracts. Note: * indicates that fatty acid and free amino acids are significantly correlated with volatile compounds (*p* < 0.05); ** indicates that they are highly significantly correlated (*p* < 0.01).

Volatile flavor compounds predominantly originate from the oxidative degradation of fatty acids (yielding aldehydes, ketones, and alcohols) as well as sulfur-containing compounds, pyrazines, and aldehydes generated through Strecker degradation of amino acids. The synergistic effects of these metabolites collectively give rise to the characteristic flavor profile of food products. This study demonstrates that SA extract significantly modulates both free amino acid composition and fatty acid profiles in mud crab muscle tissue. As illustrated in Figure 7, correlation analysis revealed that saturated fatty acids (SFA, C24:0, C16:0) and polyunsaturated fatty acids (n-3PUFA, C22:6n3, n-6PUFA, PUFA) were positively correlated with isodurene, trimethylbenzene, 2-ethyl-p-xylene, cetene, 1-octen-3-ol, pentylcyclopropane, and 2-phenylacetaldehyde, but negatively correlated with 1,3-xylene, hexadecanal, tetradecane, methional, *trans*-2-nonen-1-ol, 13-methyltetradecanal, icosanal, and nonanal. Notably, eicosapentaenoic acid (C20:5n3, EPA) and monounsaturated fatty acids (MUFA) exhibited distinct correlation patterns. Specifically, C20:5n3 showed significant positive correlations with 4′-aminoacetophenone, benzaldehyde, *p*-xylene, and 3-decanol, whereas MUFA was strongly associated with 2-methylpyrazine, pentadecane, 3-decanone, ethylbenzene, and trimethylsilyl myristate. Interestingly, both C20:5n3 and MUFA shared negative correlations with 1,3-xylene, hexadecanal, tetradecane, methional, heptadecane, and butyl isobutyl phthalate. These results suggest that *S. alterniflora* extracts modulate the synthesis and accumulation of these volatile organic compounds by altering specific fatty acid metabolic pathways. The oxidative degradation of PUFAs generates highly volatile aldehydes with distinct aromas, substantially impacting food flavor characteristics. Notably, C20:5n3 (EPA) exhibited a significant positive correlation with benzaldehyde, whereas SFAs (including C24:0), n-3PUFAs, and C22:6n3 (DHA) showed significant negative correlations with nonanal. This phenomenon is well-documented in aquatic research, where dietary modification has been shown to regulate flavor compound synthesis, particularly the crucial relationship between PUFAs and muscle flavor profiles. Supporting this, Dai et al. (2021) found that dietary PUFAs significantly affect nonanal content in Chinese mitten crab (*Eriocheir sinensis*) muscle [45]. Their findings align with our observations regarding the effects of n-3PUFA and C22:6n3 on nonanal levels, further validating the significant association between PUFAs and flavor quality in crustacean muscle tissues.

The analysis revealed significant correlations between amino acids and volatile organic compounds, with distinct association patterns emerging for different amino acid species. Specifically, Ala, His, Lys, and Val showed positive correlations with 4′-aminoacetophenone, benzaldehyde, p-xylene, and 3-decanol, but negative correlations with hexadecanal, tetradecane, and methional. In contrast, Met, Glu, and Asp exhibited positive associations with trans-2-nonen-1-ol, 13-methyltetradecanal, icosanal, and nonanal, while displaying negative associations with isodurene, trimethylbenzene, 2-ethyl-p-xylene, and cetene. Interestingly, Ile displayed an inverse correlation pattern: it was positively associated with isodurene, trimethylbenzene, 2-ethyl-p-xylene, and cetene, yet negatively correlated with icosanal, nonanal, and 13-methyltetradecanal. Arg, however, presented a more complex profile, showing positive associations with 2-nonen-1-ol, pentylcyclopropane, dimethylsilanediol, and dibutyl phthalate, alongside negative correlations with 2-nonen-1-ol, hexadecanal, tetradecane, and methional. These differential correlation patterns suggest that various amino acids participate in distinct chemical pathways, either promoting or inhibiting the formation of specific volatile compounds.

## 4. Conclusions

This study demonstrates that a 0.15% dietary supplementation level of *S. alterniflora* extract significantly enhances growth performance and improves flavor quality in mud crabs. The optimal 0.15% dosage markedly promoted crab growth, while concentrations between 0.1% and 0.15% significantly enhanced umami characteristics, optimized fatty acid profiles, and increased aldehyde compound content in crab muscle. The observed effects are likely mediated by bioactive components in the extract, including flavonoids, polysaccharides, and saponins, although the precise regulatory mechanisms require further investigation. These findings provide scientific support for developing novel aquafeed additives while establishing a sustainable utilization strategy for this invasive plant species. Furthermore, this study systematically elucidates the intricate correlation network among amino acids, fatty acids, and volatile flavor compounds, providing novel theoretical foundations for the regulation of flavor quality in aquatic animals.

## Figures and Tables

**Table 1:** Content of free amino acids in the muscle of mud crab (mg/g).

Free amino Acid Content (mg/g)	Taste	C	SA0.05	SA0.1	SA0.15	SA0.2
Asp	Umami (+)	1 ± 0.04 ^b^	0.81 ± 0.18 ^b^	1.46 ± 0.15 ^a^	1.68 ± 0.05 ^a^	1.52 ± 0.11 ^a^
Glu	Umami (+)	0.74 ± 0.03 ^c^	0.63 ± 0.1 ^c^	1.09 ± 0.18 ^b^	1.2 ± 0.04 ^b^	1.66 ± 0.11 ^a^
Ser	Sweet (+)	5.89 ± 1.63 ^a^	4.28 ± 1.06 ^a^	5.94 ± 0.6 ^a^	4.61 ± 1.39 ^a^	1.21 ± 0.87 ^b^
Gly	Sweet (+)	5.56 ± 0.57 ^ab^	4.34 ± 0.46 ^b^	5.58 ± 0.83 ^ab^	6.65 ± 1.38 ^a^	0.85 ± 0.05 ^c^
His	Bitter (−)	1.64 ± 0.15 ^b^	1.63 ± 0.24 ^b^	1.74 ± 0.24 ^b^	1.73 ± 0.09 ^b^	1.08 ± 0.04 ^a^
Arg	Bitter/Sweet (+)	7.19 ± 0.52 ^a^	5.43 ± 0.57 ^b^	5.16 ± 0.22 ^b^	7.14 ± 0.46 ^a^	2.67 ± 0.44 ^c^
Thr	Sweet (+)	1.27 ± 0.11 ^a^	1.75 ± 0.31 ^a^	1.54 ± 0.21 ^a^	1.62 ± 0.41 ^a^	1.35 ± 0.09 ^a^
Ala	Sweet (+)	3.89 ± 0.24 ^b^	3.98 ± 0.34 ^b^	4.85 ± 0.68 ^a^	5.05 ± 0.44 ^a^	2.01 ± 0.07 ^c^
Pro	Sweet/Bitter (+)	4.97 ± 0.23 ^b^	4.68 ± 0.27 ^b^	6.12 ± 0.85 ^a^	4.7 ± 0.53 ^b^	2.31 ± 0.09 ^c^
Tyr	Bitter (−)	1.63 ± 0.01 ^a^	1.4 ± 0.29 ^ab^	1.59 ± 0.25 ^a^	1.69 ± 0.25 ^a^	1.11 ± 0.1 ^b^
Val	Bitter/Sweet (−)	1.63 ± 0.12 ^bc^	1.61 ± 0.28 ^bc^	1.93 ± 0.22 ^b^	2.41 ± 0.18 ^a^	1.33 ± 0.09 ^c^
Met	Bitter/Sulfur (−)	1.36 ± 0.09 ^ab^	1.27 ± 0.27 ^b^	1.4 ± 0.23 ^ab^	1.68 ± 0.08 ^a^	1.7 ± 0.12 ^a^
Cys	Bitter/Sulfur (−)	1.04 ± 0.08 ^a^	1.31 ± 0.29 ^a^	1.11 ± 0.16 ^a^	1.19 ± 0.27 ^a^	1.37 ± 0.09 ^a^
Ile	Bitter (−)	1.9 ± 0.21 ^b^	2.68 ± 0.28 ^a^	1.6 ± 0.4 ^b^	1.85 ± 0.62 ^b^	1.49 ± 0.1 ^b^
Leu	Bitter (−)	3.02 ± 0.4 ^abc^	2.75 ± 0.31 ^bc^	3.38 ± 0.47 ^ab^	3.7 ± 0.21 ^a^	1.51 ± 0.1 ^d^
Phe	Bitter (−)	0.64 ± 0.13 ^b^	0.83 ± 0.14 ^b^	1.24 ± 0.17 ^a^	1.36 ± 0.16 ^a^	0.73 ± 0.16 ^b^
Lys	Sweet/Bitter (−)	2.36 ± 0.24 ^b^	2.64 ± 0.44 ^b^	2.79 ± 0.32 ^b^	3.62 ± 0.38 ^a^	1.66 ± 0.11 ^c^
TAA	-	45.91 ± 3.04 ^bc^	42.08 ± 3.86 ^bc^	48.58 ± 3.54 ^ab^	51.92 ± 3.35 ^a^	25.61 ± 0.84 ^d^
EAA	-	12.2 ± 0.99 ^b^	13.55 ± 1.77 ^b^	13.91 ± 1.54 ^ab^	16.25 ± 1.25 ^a^	9.78 ± 0.5 ^d^
UAA	-	1.74 ± 0.07 ^c^	1.44 ± 0.19 ^c^	2.55 ± 0.22 ^b^	2.88 ± 0.02 ^a^	3.17 ± 0.23 ^a^
SAA	-	16.61 ± 2.48 ^a^	14.36 ± 1.6 ^a^	17.93 ± 1.94 ^a^	17.94 ± 2.08 ^a^	5.44 ± 0.31 ^b^
BAA	-	12.85 ± 0.94 ^b^	13.56 ± 1.72 ^b^	14.3 ± 1.61 ^ab^	16.37 ± 1.09 ^a^	8.94 ± 0.4 ^c^

Note: FREE AMINO ACID indicates free amino acids. (+) Indicates that this amino acid is pleasant, (−) indicates that this amino acid is unpleasant. EAA (essential amino acid); TAA (total free amino acid); UAA (umami amino acid); SAA (sweet amino acid); BAA (Bitter amino acid). Data are presented as the mean ± standard deviation of three replicates. Additional letters in the same row indicate a significant difference (*p* < 0.05). “-” indicates could not be determined. Same below.

**Table 2:** Content of nucleotides in the muscle of mud crab (mg/100 g).

Flavor Nucleotides (mg/100 g)	C	SA0.05	SA0.1	SA0.15	SA0.2
AMP	27.42 ± 1.05 ^cd^	51.03 ± 7.42 ^b^	23.62 ± 0.91 ^d^	64.64 ± 9.05 ^a^	36.21 ± 2.8 ^c^
GMP	8.53 ± 1.58 ^a^	5.86 ± 0.62 ^b^	7.83 ± 1.03 ^ab^	7.27 ± 0.82 ^ab^	7.59 ± 1.63 ^ab^
IMP	62.79 ± 6.19 ^d^	53.85 ± 4.68 ^d^	143 ± 10.75 ^b^	181.35 ± 4.64 ^a^	98.34 ± 4.94 ^c^

Data are presented as the mean ± standard deviation of three replicates. Additional letters in the same row indicate a significant difference (*p* < 0.05).

**Table 3:** Content of fatty acids in the muscle of mud crab paramamosain (mg/g).

Fatty Acid (mg/100 g)	C	SA0.05	SA0.1	SA0.15	SA0.2
C4:0	1.25 ± 0.19 ^a^	1.41 ± 0.4 ^a^	1.22 ± 0.05 ^ab^	1.32 ± 0.33 ^ab^	0.88 ± 0.1 ^b^
C6:0	1.89 ± 0.3 ^c^	3.24 ± 0.2 ^a^	1.26 ± 0.1 ^d^	1.36 ± 0.1 ^d^	2.56 ± 0.35 ^b^
C8:0	0.35 ± 0.021 ^b^	0.45 ± 0.1 ^b^	0.31 ± 0.14 ^b^	0.54 ± 0.1 ^a^	0.75 ± 0.21 ^a^
C10:0	0.93 ± 0.04 ^ab^	0.65 ± 0.08 ^b^	0.7 ± 0.11 ^b^	0.77 ± 0.15 ^ab^	1.02 ± 0.27 ^a^
C11:0	11.14 ± 0.98 ^a^	8.17 ± 1.61 ^b^	8.28 ± 1.83 ^b^	7.79 ± 0.7 ^b^	6.25 ± 0.82 ^b^
C12:0	0.73 ± 0.06 ^a^	0.34 ± 0.02 ^bc^	0.2 ± 0.04 ^c^	0.25 ± 0.15 ^c^	0.45 ± 0.11 ^b^
C13:0	0.8 ± 0.02 ^a^	0.73 ± 0.13 ^a^	0.53 ± 0.07 ^b^	0.64 ± 0.05 ^ab^	0.71 ± 0.11 ^a^
C14:0	4.42 ± 1.09 ^a^	3.91 ± 0.8 ^ab^	2.78 ± 0.67 ^bc^	2.68 ± 0.32 ^bc^	1.57 ± 0.29 ^c^
C14:1	0.27 ± 0.11 ^b^	0.51 ± 0.05 ^a^	0.24 ± 0.01 ^b^	0.23 ± 0.05 ^b^	0.39 ± 0.17 ^ab^
C:15:0	0.46 ± 0.1 ^a^	0.58 ± 0.08 ^b^	0.47 ± 0.13 ^b^	0.6 ± 0.08 ^b^	0.32 ± 0.13 ^b^
C15:1	0.5 ± 0.18 ^ab^	0.26 ± 0.06 ^b^	0.27 ± 0.16 ^b^	0.33 ± 0.09 ^ab^	0.54 ± 0.08 ^a^
C16:0	44.9 ± 1.91 ^a^	37.54 ± 1.07 ^b^	37.57 ± 1.12 ^b^	32.64 ± 2.26 ^c^	24.19 ± 1.4 ^d^
C16:1	1.44 ± 0.03 ^a^	1.01 ± 0.15 ^b^	0.94 ± 0.12 ^b^	0.84 ± 0.11 ^b^	0.47 ± 0.14 ^c^
C17:0	0.56 ± 0.07 ^a^	0.67 ± 0.06 ^a^	0.53. ± 0.16 ^a^	0.69 ± 0.08 ^a^	0.59 ± 0.08 ^a^
C17:1	18.48 ± 2.04 ^ab^	16.35 ± 0.69 ^b^	19.48 ± 1.86 ^a^	16.38 ± 0.78 ^b^	13.6 ± 1.09 ^c^
C18:0	4.99 ± 1.19 ^a^	5.59 ± 0.74 ^a^	5.02 ± 0.37 ^a^	4.57 ± 0.52 ^a^	3.21 ± 0.54 ^b^
C18:1n9t	15.93 ± 4.27 ^b^	19.57 ± 1.01 ^b^	24.47 ± 2.74 ^a^	19.3 ± 1.47 ^b^	11.38 ± 1.12 ^c^
C18:1n9c	0.52 ± 0.01 ^a^	0.67 ± 0.06 ^a^	0.49 ± 0.17 ^a^	0.69 ± 0.14 ^a^	0.5 ± 0.2 ^a^
C18:2n6t	35.82 ± 2.9 ^a^	34.57 ± 1.3 ^a^	28.91 ± 3.57 ^b^	30.73 ± 3.34 ^ab^	19.47 ± 2.09 ^c^
C18:2n6c	0.42 ± 0.08 ^a^	0.34 ± 0.06 ^a^	0.39 ± 0.05 ^a^	0.39 ± 0.03 ^a^	0.31 ± 0.04 ^a^
C20:0	0.96 ± 0.05 ^b^	1.65 ± 0.05 ^a^	1.61 ± 0.25 ^a^	1.6 ± 0.07 ^a^	1.17 ± 0.16 ^b^
C18:3n6	1.21 ± 0.35 ^a^	1.42 ± 0.07 ^a^	1.18 ± 0.13 ^a^	0.8 ± 0.03 ^b^	0.46 ± 0.14 ^c^
C20:1	2.77 ± 0.65 ^a^	1.75 ± 0.2 ^bc^	2.07 ± 0.26 ^b^	1.37 ± 0.21 ^cd^	0.74 ± 0.19 ^d^
C18:3n3	0.4 ± 0.14 ^a^	0.45 ± 0.11 ^a^	0.46 ± 0.15 ^a^	0.41 ± 0.09 ^a^	0.44 ± 0.06 ^a^
C21:0	3.08 ± 0.85 ^a^	2.7 ± 0.22 ^ab^	2.52 ± 0.38 ^ab^	2.13 ± 0.19 ^bc^	1.43 ± 0.22 ^c^
C20:2	0.41 ± 0.66 ^a^	0.2 ± 0.06 ^b^	0.32 ± 0.13 ^ab^	0.39 ± 0.08 ^a^	0.21 ± 0.09 ^b^
C22:0	0.26 ± 0.07 ^a^	0.21 ± 0.06 ^a^	0.32 ± 0.06 ^a^	0.26 ± 0.08 ^a^	0.28 ± 0.03 ^a^
C20:3n6	0.51 ± 0.65 ^b^	0.47 ± 0.07 ^bc^	0.28 ± 0.13 ^cd^	0.74 ± 0.17 ^a^	0.21 ± 0.09 ^d^
C22:1n9	0.5 ± 0.06 ^a^	0.29 ± 0.11 ^bc^	0.17 ± 0.03 ^c^	0.33 ± 0.09 ^b^	0.25 ± 0.06 ^bc^
C20:3n3	0.29 ± 0.04 ^a^	0.36 ± 0.03 ^b^	0.36 ± 0.1 ^b^	0.39 ± 0.12 ^b^	0.37 ± 0.07 ^b^
C20:4n6	2.76 ± 0.93 ^b^	3.37 ± 1.32 ^b^	5.22 ± 0.47 ^a^	3.84 ± 0.28 ^ab^	3.01 ± 0.94 ^b^
C23:0	0.77 ± 0.18 ^a^	0.54 ± 0.15 ^ab^	0.57 ± 0.13 ^ab^	0.46 ± 0.06 ^b^	0.37 ± 0.08 ^b^
C22:2	0.08 ± 0.01 ^a^	0.06 ± 0.01 ^b^	0.06 ± 0.01 ^b^	0.06 ± 0.01 ^b^	0.05 ± 0.02 ^b^
C24:0	84.72 ± 5.3 ^a^	80 ± 2.89 ^a^	67.28 ± 3.21 ^b^	60.14 ± 1.97 ^b^	37.03 ± 5.68 ^c^
C20:5n3(EPA)	24.96 ± 3.2 ^c^	30.86 ± 0.7 ^b^	31.69 ± 3.49 ^b^	43.56 ± 3.74 ^a^	12.22 ± 0.9 ^d^
C24:1	0.23 ± 0.04 ^a^	0.17 ± 0.02 ^ab^	0.13 ± 0.01 ^b^	0.18 ± 0.01 ^ab^	0.13 ± 0.02 ^b^
C22:6n3(DHA)	43.01 ± 2.3 ^a^	36.25 ± 3.48 ^b^	23.92 ± 0.89 ^c^	21.15 ± 0.42 ^c^	12.97 ± 1.92 ^d^
SFA	163.08 ± 5.97 ^a^	149.25 ± 3.81 ^b^	131.77 ± 6.09 ^c^	119.09 ± 3.52 ^d^	83.8 ± 7.21 ^e^
MUFA	40.67 ± 5.77 ^b^	40.62 ± 0.65 ^b^	48.31 ± 4.86 ^a^	37.68 ± 2.27 ^b^	28.04 ± 1.54 ^c^
PUFA	111.1 ± 1.47 ^a^	108.95 ± 5.74 ^a^	93.41 ± 8.21 ^b^	102.97 ± 5.77 ^ab^	50.12 ± 3.42 ^c^
n-3 PUFA	68.98 ± 5.37 ^a^	67.94 ± 3.26 ^a^	56.45 ± 4.64 ^b^	65.52 ± 3.59 ^a^	26.01 ± 1.04 ^c^
n-6 PUFA	40.75 ± 4.1 ^a^	40.2 ± 2.78 ^a^	35.99 ± 4.27 ^a^	36.52 ± 3.3 ^a^	23.47 ± 2.5 ^b^
n-3/n-6 PUFA	1.71 ± 0.32 ^a^	1.69 ± 0.08 ^a^	1.57 ± 0.14 ^a^	1.8 ± 0.14 ^a^	1.11 ± 0.09 ^b^

Data are presented as the mean ± standard deviation of three replicates. Additional letters in the same row indicate a significant difference (*p* < 0.05).

**Table 4:** Content of volatile compounds in the muscle of mud crab paramamosain (ng/g).

Volatile Compound	C	SA0.05	SA0.1	SA0.15	SA0.2
Aldehydes (9)					
Benzaldehyde	99.09 ± 3.9 ^c^	120.72 ± 12.18 ^b^	131.39 ± 6.86 ^b^	182.62 ± 12.64 ^a^	123.8 ± 13.5 ^b^
2-Phenylacetaldehyde	30.06 ± 1.3 ^c^	78.9 ± 6.41 ^a^	60.11 ± 10.45 ^b^	60.7 ± 9.77 ^b^	16.65 ± 2.86 ^d^
Methional	17.66 ± 3.92 ^b^	14.61 ± 2.2 ^bc^	9.02 ± 2.26 ^cd^	5.78 ± 1.16 ^d^	34.43 ± 7.47 ^a^
Nonanal	115.49 ± 10.08 ^c^	87.07 ± 3.49 ^d^	219.94 ± 4.81 ^a^	165.97 ± 7.1 ^b^	172.06 ± 9.64 ^b^
Phenylglyoxal	2.49 ± 0.18	NF	NF	3.22 ± 0.23	1.83 ± 0.13
Hexadecanal	NF	5 ± 1.8	NF	8.32 ± 2.89	18.16 ± 0.9
Stearaldehyde	8.95 ± 1.42	NF	NF	9.58 ± 1.74	8.46 ± 1.32
Icosanal	NF	NF	NF	25.27 ± 5.9	19.96 ± 1.89
13-Methyltetradecanal	NF	NF	NF	23.18 ± 6.78	11.41 ± 2.29
Total	273.76 ± 16.93 ^d^	306.31 ± 1.94 ^c^	420.47 ± 3.37 ^b^	484.67 ± 14.53 ^a^	406.73 ± 1.8 ^b^
Alcohols (4)					
2-Nonen-1-ol	4.69 ± 1.24	NF	NF	3.07 ± 0.28	1.53 ± 0.39
3-Decanol	NF	44.22 ± 9.93	138.83 ± 13.64	127.3 ± 15.64	23.04 ± 4.83
1-Octen-3-ol	147.4 ± 1.01 ^a^	121.67 ± 1.52 ^b^	113.08 ± 0.99 ^c^	111.54 ± 0.1 ^c^	86.8 ± 3.81 ^d^
Trans-2-Nonen-1-ol	NF	NF	NF	2.72 ± 0.28	1.59 ± 0.2
Total	152.09 ± 2.24 ^b^	165.89 ± 11.02 ^b^	251.91 ± 12.92 ^a^	244.63 ± 14.16 ^a^	112.97 ± 1.67 ^d^
Ketones (2)					
4′-Aminoacetophenone	1.32 ± 0.16 ^c^	1.34 ± 0.19 ^c^	2.58 ± 0.11 ^a^	2.39 ± 0.15 ^a^	1.63 ± 0.07 ^b^
3-Decanone	39.94 ± 7.26 ^bc^	44.22 ± 9.93 ^b^	85.83 ± 8.52 ^a^	77.3 ± 15.64 ^a^	23.04 ± 4.83 ^c^
Total	41.26 ± 7.24 ^b^	45.57 ± 10.01 ^b^	88.08 ± 8.57 ^a^	79.88 ± 15.53 ^a^	24.67 ± 4.76 ^b^
Esters (4)					
Butyl isobutyl phthalate	4.9 ± 2.4	7.71 ± 1.07	NF	NF	6.79 ± 1.53
Dibutyl phthalate	16.66 ± 1.31 ^b^	7.88 ± 1.14 ^c^	6.9 ± 1.31 ^c^	23.89 ± 2.26 ^a^	7.59 ± 2.4 ^c^
Trimethylsilyl Hexadecanoate	NF	NF	26.78 ± 2.74	NF	5.2 ± 1.25
Trimethylsilyl myristate	NF	NF	35.29 ± 8.34	13.09 ± 3.35	NF
Total	21.56 ± 3.56 ^c^	15.59 ± 2.12 ^c^	68.98 ± 6.96 ^a^	36.99 ± 5.62 ^b^	19.58 ± 1.83 ^c^
Hydrocarbons (8)					
Hexadecane	17.67 ± 5.02	17.84 ± 5.77	NF	28.45 ± 3.65	14.94 ± 1.71
Pentadecane	15.35 ± 3.78 ^b^	32.76 ± 5.39 ^a^	34.21 ± 6.09 ^a^	33.86 ± 8.29 ^a^	15.72 ± 4.72 ^b^
Tetradecane	14.44 ± 4.58	16.05 ± 4.55	22.82 ± 3.18	NF	53.81 ± 2.39
Heptadecane	62.65 ± 6.29 ^a^	31.94 ± 3.53 ^b^	33.35 ± 6.91 ^b^	15.28 ± 2.87 ^c^	64.21 ± 1.88 ^a^
Dimethylsilanediol	13.34 ± 2.05	5.86 ± 2.56	NF	21.7 ± 1.83	1.82 ± 0.54
Cyclomethicone 6	NF	5.22 ± 1.97	3.76 ± 2.83	6.6 ± 0.55	2.59 ± 1.04
Pentylcyclopropane	20.74 ± 2.46	7.12 ± 4.41	NF	13.54 ± 6.99	4.24 ± 2.59
Cetene	5.15 ± 0.65	10.47 ± 1.71	NF	NF	NF
Total	149.37 ± 18.1 ^ab^	127.27 ± 13.8 ^ab^	94.16 ± 9.63 ^cd^	119.45 ± 17.56 ^c^	157.35 ± 5.53 ^a^
Aromatics (7)					
Trimethylbenzene	14.82 ± 3.6	10.53 ± 1.56	NF	NF	NF
Isodurene	13.53 ± 3.21	11.79 ± 2.34	NF	NF	NF
1,3-Xylene	16.15 ± 2.41			21.37 ± 0.62	27.65 ± 1.27
p-Xylene	5.17 ± 3.56 ^c^	8.28 ± 1.92 ^c^	20.15 ± 4.28 ^b^	36.51 ± 4.04 ^a^	7.38 ± 0.72 ^c^
2-Ethyl-p-xylene	10.06 ± 4.01	5.83 ± 1.28	NF	NF	NF
O-Xylene	5.06 ± 3.84 ^c^	14.73 ± 4.82 ^b^	24.29 ± 4.52 ^a^	14.09 ± 4.34 ^b^	13.49 ± 5.49 ^b^
Ethylbenzene	7.86 ± 4.36	NF	28.36 ± 0.79	16.84 ± 3.53	2.81 ± 1.13
Total	72.67 ± 11.74 ^b^	51.18 ± 2.68 ^c^	72.81 ± 5.16 ^b^	88.83 ± 5.77 ^a^	51.33 ± 5.1 ^c^
Other (2)					
Indole	NF	44.91 ± 2.32	NF	44.41 ± 1.69	NF
2-Methylpyrazine	NF	17.2 ± 5.07	25.16 ± 1.83	13.34 ± 1.04	NF
Total	NF	62.11 ± 4.11	25.16 ± 1.83	57.75 ± 2.11	NF

Data are presented as the mean ± standard deviation of three replicates. Additional letters in the same row indicate a significant difference (*p* < 0.05). NF: not found.

## Data Availability

The original contributions presented in this study are included in the article. Further inquiries can be directed to the corresponding authors.

## Supplementary Materials

The following supporting information can be downloaded at: https://www.mdpi.com/article/10.3390/foods14244176/s1, Table S1: Formulation and proximate composition of the experimental diets.
