# Supplementary material for: Effects of Spartina alterniflora Extract on Growth Performance and Flavor Quality in Mud Crab (Scylla paramamosain)
Source: Foods. 2025 Dec 5;14(24):4176. doi: 10.3390/foods14244176 (PMC12731809; doi:10.3390/foods14244176)
Supplement: Supplementary file 1 [file foods-14-04176-s001.zip › foods-3936045-supplementary/TableS1 Formulation and proximate composition of the experimental diets.docx]

**Table S1.** Formulation and proximate composition of the experimental diets

| **Ingredients，%** | **C** | **SA0.05** | **SA0.1** | **SA0.15** | **SA0.2** |
| --- | --- | --- | --- | --- | --- |
| Imported fish | 48 | 48 | 48 | 48 | 48 |
| Soybean meal | 19 | 19 | 19 | 19 | 19 |
| High-precision flour | 20 | 20 | 20 | 20 | 20 |
| Fish oil | 1 | 1 | 1 | 1 | 1 |
| Soybean oil  Yeast extract paste  Calcium biphosphate  Common salt  Premix  Molting hormone  Soyabean lecithin  Vitamin C  Microcrystalline cellulose  Spartina alterniflora extract | 1  2  2  1  2  0.5  1  0.5  2  0 | 1  2  2  1  2  0.5  1  0.5  1.95  0.05 | 1  2  2  1  2  0.5  1  0.5  1.9  0.1 | 1  2  2  1  2  0.5  1  0.5  1.85  0.15 | 1  2  2  1  2  0.5  1  0.5  1.8  0.2 |
| Total | 100 | 100 | 100 | 100 | 100 |
| Nutritional components (Dry Weight) | | | | | |
| crude protein | 46.83 | 46.31 | 46.60 | 46.67 | 46.65 |
| crude fat | 8.11 | 8.19 | 8.21 | 8.12 | 8.22 |

Note : C, SA0.05,SA0.1,SA0.15 and SA0.2 represent the control group without S.alterniflora extract and the treatment group with SA extract concentration of 0.05 %, 0.1 %, 0.15 % and 0.2 %, respectively.
